# Keeping inflammation at bay

**DOI:** 10.7554/eLife.02583

**Published:** 2014-03-25

**Authors:** David Wallach, Andrew Kovalenko

**Affiliations:** 1 **David Wallach** is an *eLife* reviewing editor, and is in the Department of Biological Chemistry, The Weizmann Institute of Science, Rehovot, Israeld.wallach@weizmann.ac.il; 2 **Andrew Kovalenko** is in the Department of Biological Chemistry, The Weizmann Institute of Science, Rehovot, Israel

**Keywords:** apoptosis, macrophage, inflammation, adenosine receptors, mouse

## Abstract

Cells dying by apoptosis can trigger an anti-inflammatory gene response in other cells by releasing a compound called adenosine monophosphate.

**Related research article** Yamaguchi H, Maruyama T, Urade Y, Nagata S. 2014. Immunosuppression via adenosine receptor activation by adenosine monophosphate released from apoptotic cells. *eLife* **3**:e02172. doi: 10.7554/eLife.02172

**Image** AMP molecules released by apoptotic cells can trigger an anti-inflammatory response in phagocytes.

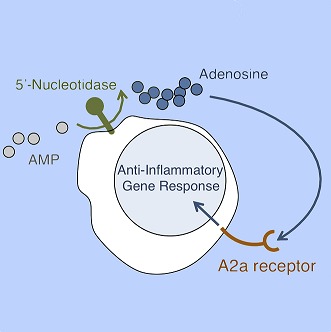

The cells in our bodies are genetically programmed to undergo a natural process of self-destruction called apoptosis, after which the dying cell is removed by cells that have the ability to engulf them (‘phagocytes’). The membrane of the dying cell is still intact as it is engulfed by the phagocyte, so its contents do not come into contact with other nearby cells. Apoptosis does not trigger inflammation, whereas another form of cell death called necrosis—in which the cell membrane is ruptured—is often associated with inflammation (Kerr et al., 1972).

Necrosis causes inflammation because some components of the dying cell that are capable of triggering inflammation come into contact with healthy cells nearby (Rock and Kono, 2008). At first it was assumed that the only reason why apoptosis did not cause inflammation was that all the contents of the dying cell remained inside the membrane and the phagocyte. However, it was later discovered that apoptosis can actually block inflammation *(*Voll et al., 1997; Fadok et al., 1998). Initial observations suggested that this anti-inflammatory effect is triggered when the phagocytes are exposed to phosphatidylserine—a molecule on the surface of apoptotic cells that has a central role in phagocytosis (Huynh et al., 2002). It seemed, therefore, that these anti-inflammatory changes could be induced only in cells intimately associated with the dying cell (Figure 1A).

**Figure 1.**
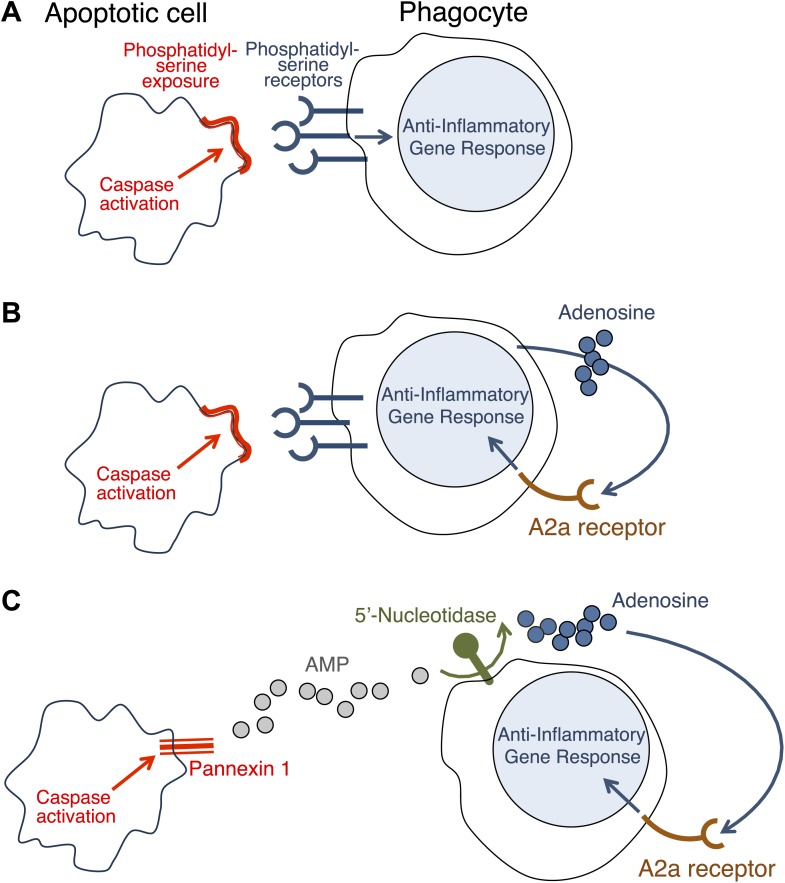
How do apoptotic cells trigger an anti-inflammatory response in phagocytes? (**A**) Phosphatidylserine molecules on the surface of an apoptotic cell can bind to phosphatidylserine receptors on the surface of a phagocyte and previously it was suggested that this triggered an anti-inflammatory gene response. (**B**) It was also suggested that the direct apoptotic cell–phagocyte interaction shown in **A** also results in the release of adenosine by the phagocyte: this adenosine can bind to A2a receptors on the surface of the phagocyte and trigger an anti-inflammatory gene response. (**C**) Yamaguchi et al. found that the apoptotic cell releases a molecule called adenosine monophosphate (AMP) that is converted to adenosine by a 5′-nucleotidase on the surface of the phagocyte. The adenosine can then trigger an anti-inflammatory gene response by binding to A2a receptors. Enzymes called caspases play a central role in apoptosis in a variety of ways. The action of these caspases is required for the exposure of phosphatidylserine on the surface of the apoptotic cells (**A** and **B**); they also activate a channel protein called pannexin-1 to allow the release of AMP (**C**).

Now, in *eLife*, Shigekazu Nagata and co-workers at Kyoto University and the Osaka Bioscience Institute—including Hiroshi Yamaguchi as first author—report that apoptotic cells release a molecule called adenosine that can activate an anti-inflammatory gene response in phagocytes (Yamaguchi et al., 2014). They have also shown that adenosine activates this response by stimulating the A2a adenosine receptor in phagocytes.

Similar results have been reported before (Sitkovsky and Ohta, 2005; Köröskényi et al., 2011), but it had been thought that the adenosine was generated by the phagocytes as a consequence of their uptake of the apoptotic cells (Figure 1B). Yamaguchi et al. now show that the adenosine comes from the apoptotic cells themselves, with the phagocytes having only a secondary role in its production. The first step involves enzymes called caspases—which have a central role in apoptosis—cleaving a membrane channel protein called pannexin-1 in the dying cells, and thereby activating it. This results in the release of adenosine monophosphate (AMP) from the dying cells. A 5′-nucleotidase expressed by the phagocytes then removes a phosphate group from the AMP to yield adenosine. The adenosine then binds to the A2a receptor on the phagocytes to trigger an anti-inflammatory gene response (Figure 1C).

Adenosine is not the only soluble molecule released by apoptotic cells to perform a specific role. For example, various other molecules—including lysophosphatidylcholine and the nucleotides ATP and UTP—act as ‘find me’ signals that attract phagocytes towards apoptotic cells (Hochreiter-Hufford and Ravichandran, 2013). Another example is an iron-binding glycoprotein called lactoferrin that inhibits the translocation of certain white blood cells, thereby apparently contributing to the anti-inflammatory effect of apoptosis (Bournazou et al., 2009).

To what extent do the soluble molecules released by apoptotic cells have an effect on cells remote from the site of death? And how does the contribution of these molecules to the anti-inflammatory consequences of apoptosis compare with the contribution that results from direct contact between the dying cell and the cell engulfing it? Nagata and co-workers report that in a mouse model of inflammation (zymosan-induced peritonitis), deletion of either the *Pannexin-1* gene or the *A2a* gene prolongs the inflammation. These findings support the notion that (in this experimental model) adenosine derived from apoptotic cells contributes significantly to the restriction of inflammation. More precise cell-type-specific targeting of these molecules (and other molecules that have anti-inflammatory effects) should lead to an improved understanding of their relative contributions to immune regulation in specific pathological situations.
